# Metabolism of pancreatic cancer: paving the way to better anticancer strategies

**DOI:** 10.1186/s12943-020-01169-7

**Published:** 2020-03-02

**Authors:** Cheng Qin, Gang Yang, Jinshou Yang, Bo Ren, Huanyu Wang, Guangyu Chen, Fangyu Zhao, Lei You, Weibin Wang, Yupei Zhao

**Affiliations:** 1 Department of General Surgery, Peking Union Medical College Hospital, Chinese Academy of Medical Sciences, Peking Union Medical College, Beijing, 100730 PR China; 2 Department of General Surgery, Peking Union Medical College Hospital, Chinese Academy of Medical Sciences, Peking Union Medical College, Beijing, 100023 PR China

**Keywords:** Pancreatic cancer, Metabolism, Chemoresistance, Gemcitabine, Radioresistance, Immunosuppression, Clinical trials

## Abstract

Pancreatic cancer is currently one of the most lethal diseases. In recent years, increasing evidence has shown that reprogrammed metabolism may play a critical role in the carcinogenesis, progression, treatment and prognosis of pancreatic cancer. Affected by internal or external factors, pancreatic cancer cells adopt extensively distinct metabolic processes to meet their demand for growth. Rewired glucose, amino acid and lipid metabolism and metabolic crosstalk within the tumor microenvironment contribute to unlimited pancreatic tumor progression. In addition, the metabolic reprogramming involved in pancreatic cancer resistance is also closely related to chemotherapy, radiotherapy and immunotherapy, and results in a poor prognosis. Reflective of the key role of metabolism, the number of preclinical and clinical trials about metabolism-targeted therapies for pancreatic cancer is increasing. The poor prognosis of pancreatic cancer patients might be largely improved after employing therapies that regulate metabolism. Thus, investigations of metabolism not only benefit the understanding of carcinogenesis and cancer progression but also provide new insights for treatments against pancreatic cancer.

## Background

Pancreatic cancer is one of the most aggressive diseases; it has a poor prognosis, and its five-year survival rate remains lower than 9% despite decades of continuous efforts. According to recent data, pancreatic cancer is the fourth and sixth leading cause of cancer-related mortality in the U.S. and China, respectively [1, 2]. Furthermore, it is predicted to be the second leading cause of cancer-related deaths in the U.S. in 2030 [3]. Surgery remains the only way to cure pancreatic cancer. However, because most patients are diagnosed with a nonresectable disease due to the lack of symptoms in the early stage, only up to 20% of patients have the opportunity to receive initial surgical resection [4]. Even for patients who undergo a successful operation, over 80% of them still eventually develop local recurrence or metastases [5]. Therefore, in addition to surgery, comprehensive treatment following multidisciplinary management should be given more attention. Indeed, there remain many challenging problems. Chemotherapy is still recommended as the primary treatment for patients who have nonresectable pancreatic cancer and patients who undergo resection. Depending on different patient statuses, there are several distinct recommended chemotherapy regimens, which mainly include gemcitabine, FOLFIRINOX, and albumin-bound paclitaxel. However, the rapid and common development of chemoresistance usually leads to poor prognosis [6]. Although radiation is another relatively well-established anticancer method, it is currently regarded as a palliative way to relieve pain caused by advanced pancreatic cancer [7]. In addition to chemotherapy and radiotherapy, immunotherapy and targeted therapy are emerging as remarkable anticancer strategies [8–10]. Nevertheless, many successful immunotherapies against other cancer types are not as effective in pancreatic cancer treatment [11, 12], and most clinical trials focusing on targeted therapy fail to show satisfying outcomes [13]. Therefore, breakthroughs in pancreatic cancer treatment are needed.

In pancreatic cancer cells, several genetic alterations are considered to be the basis for pancreatic cancer progression and its dismal prognosis; these alterations include oncogenic *KRAS* mutations, which occur in over 90% of cases, and inactivating mutations in suppressor genes such as *TP53*, *SMAD4*, and *CDKN2A* [14]. Moreover, the aforementioned dilemma in comprehensive treatments is also largely determined by other biological features, such as extensive dense desmoplasia, hypoperfusion and an immunosuppressive microenvironment [15]. Additionally, many recent reports have indicated that distinct cancer metabolism is important for restricting the therapeutic effect.

Reprogrammed cellular energy metabolism, one of the emerging hallmarks of cancer [16], has been refocused over the past decade [17]. Cancer cells rewire many metabolic pathways to facilitate their survival, unlimited cell growth, and division. In addition, they also rely on extensive metabolic interactions with other nonmalignant cells and with the extracellular matrix (ECM) within the tumor microenvironment [18, 19]. Beyond the tissue level, the local tumor can affect host metabolism via cachexia, impairing antitumor immunity [20]. Interestingly, several recent studies also demonstrated that metabolic alterations can promote pancreatic tumorigenesis and metastasis through epigenetic regulation [21, 22], emphasizing the vital role of metabolism in pancreatic cancer development. Furthermore, many studies clearly showed that pancreatic tumor metabolism is closely associated with chemoresistance [23], radioresistance [24] and immunosuppression [25]. Recently, pancreatic cancer was also stratified into different metabolic subgroups (quiescent, glycolytic, cholesterogenic and mixed), which could predict different prognoses and responses to therapy [26, 27]. Therefore, the metabolic features of pancreatic cancer provide attractive therapeutic opportunities for novel and personalized treatments [27, 28].

## Metabolic features of pancreatic cancer

Although reprogrammed metabolism is a general characteristic of cancer, different cancers show distinct metabolic addictions, which are mainly determined by their specific genetic mutations, tissue of origin or tumor microenvironment [29, 30]. Even in the same pancreatic cancer patient, the primary tumor and metastatic lesions exhibit relatively different metabolic gene expression [31]. Therefore, metabolic alteration of pancreatic cancer is a collective scenario mediated by multiple factors. In addition to the genomic characterization of pancreatic cancer cells [32], there is a complex and harsh microenvironment within the pancreatic tumor. The dense stroma results in elevated solid stress and interstitial fluid pressure that compress the vasculature, leading to hypoperfusion [33]. However, cancer cells exhibit extraordinary growth advantages in relatively hypoxic and nutrient-poor niches. They survive and thrive mainly in three ways: (1) Reprogramming intracellular energy metabolism of nutrients, including glucose, amino acids, and lipids. (2) Improving nutrient acquisition by scavenging and recycling. (3) Conducting metabolic crosstalk with other components within the microenvironment [34].

### Intracellular metabolism

**Fig. 1:**
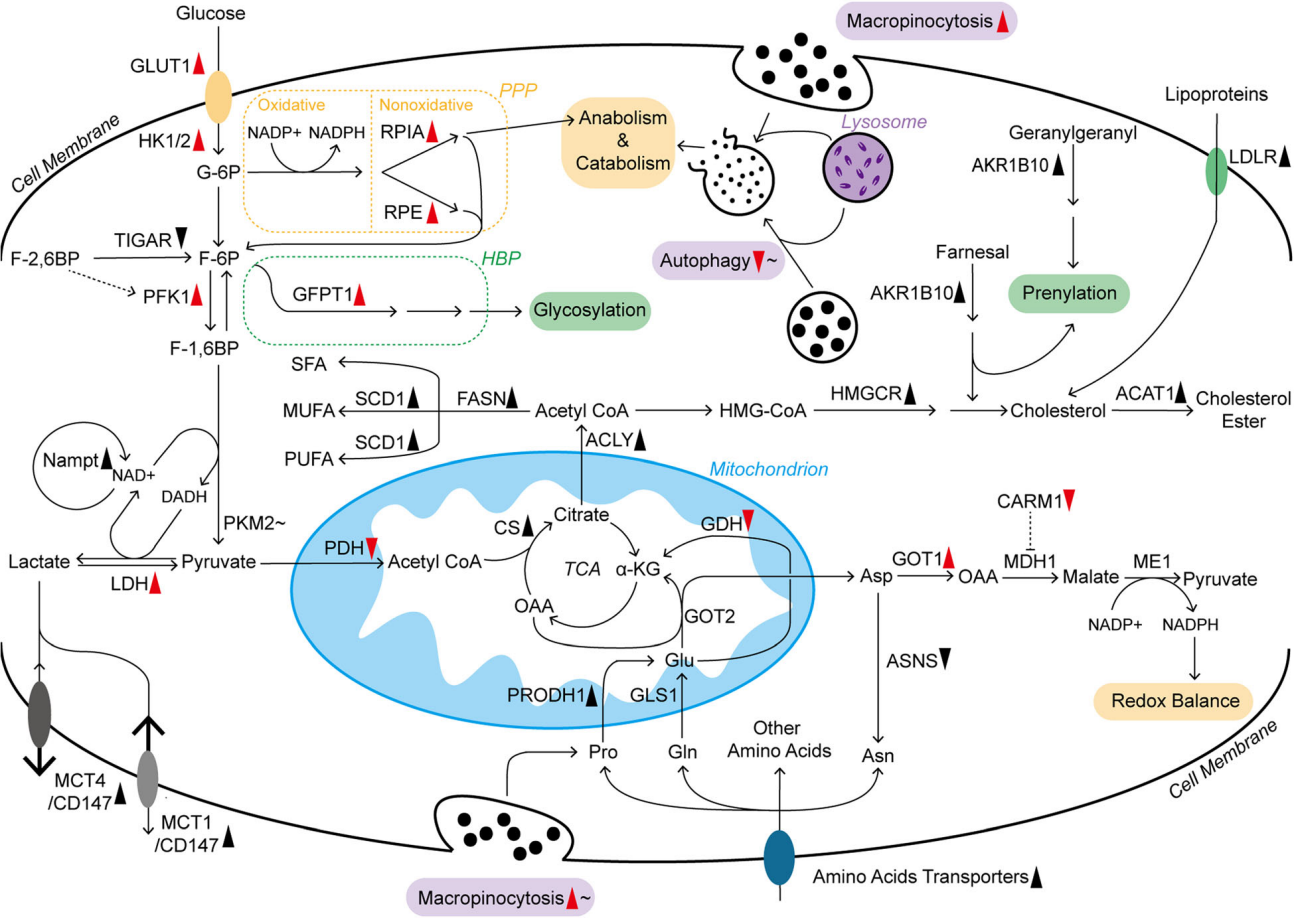
The landscape of metabolic pathways in pancreatic cancer cells. The metabolism of glucose, amino acids and lipids is largely reprogrammed, which is mainly due to changes in key enzymes and transporters. Furthermore, some of them are closely regulated by oncogenic KRAS. Additionally, micropinocytosis and autophagy are also promoted by mutant KRAS, but they are controlled by other regulatory mechanisms within pancreatic cancer cells as well. Long solid arrows imply shifts or bioconversions. The dotted arrow means positive regulation, whereas the blunt end means negative regulation. Red arrowheads following the enzymes, transporters, and processes represent the effects induced by mutant KRAS: upward means upregulation; downward means downregulation. The black symbols represent the changes induced by other or unknown reasons. In addition, those following tildes indicate that they are dually regulated under different conditions. ACLY, ATP citrate lyase; ASNS, asparagine synthetase; CARM1, coactivator-associated arginine methyltransferase 1; CS, citrate synthetase; F-6P, fructose 6-phosphate; F-1,6BP, fructose 1,6-bisphosphate; F-2,6BP, fructose 2,6-bisphosphate; GFPT1, glutamine:fructose 6-phosphate amidotransferase 1; G-6P, glucose 6-phosphate; HK1/2, hexokinase 1/2; HMGCR, 3-hydroxy-3-methylglutaryl coenzyme A reductase; HMG-CoA, 3-hydroxy-3-methylglutaryl coenzyme A; ME1, malic enzyme; MUFA, monounsaturated fatty acid; PFK1, phosphofructokinase 1; PRODH1, proline oxidase; PUFA, polyunsaturated fatty acid; RPE, ribulose-5-phosphate epimerase; SCD1, stearoyl-CoA desaturase; SFA, saturated fatty acid; TCA, tricarboxylic acid

In the 1920s, Otto Warburg’s pioneering work demonstrated that tumor cells consume more glucose than normal cells. They subsequently turn most glucose-derived carbon into lactate even in the presence of sufficient oxygen. This process is named aerobic glycolysis or the Warburg effect [35]. It indeed provides some tangible advantages to cancer cells. First, compared with oxidative phosphorylation (OXPHOS), ample glycolytic flux achieves a higher rate of ATP production [36]. Second, it provides tumor cells with plenty of intermediates required for rapid and vast biosynthesis with a proper ATP/ADP ratio. Third, it plays an important role in maintaining redox balance and modulating chromatin state. Fourth, it creates a low immunity microenvironment and enhances cancer cell invasion [37]. Since Warburg’s initial publications, many studies have been conducted to uncover the metabolism of tumors. Cancers with different tissue origins exhibit distinct metabolic changes, even driven by the same oncogenes [38]. For pancreatic cancer cells, genetic mutations and stromal cues are thought to drive heterogeneous metabolic phenotypes [39–43], which mainly include the Warburg, reverse Warburg, lipid-dependence, and glutaminolysis phenotypes [44]. Therefore, pancreatic cancer cells exhibit complex and heterogeneous reprogramming of glucose, amino acid and lipid metabolism (Fig. 1).

#### Glucose

Glucose metabolism is relatively well documented in the rewired metabolic network. In the process of elevated aerobic glycolysis in pancreatic cancer cells, the expression of glucose transporter 1 (GLUT1) and its translocation to the cancer cell membrane are promoted, increasing glucose uptake [39, 45]. In addition to absorption, pancreatic cancer also shows upregulated expression of many genes encoding rate-limiting glycolytic enzymes, such as hexokinase 1/2, phosphofructokinase 1 and lactate dehydrogenase A (LDHA, the subunit of LDH), enhancing the Warburg effect and glycolytic flux to lactate [39]. In contrast to glycolysis, phosphorylated pyruvate dehydrogenase kinase 1 (PDHK1) inhibits the pyruvate dehydrogenase (PDH) complex, suppressing mitochondrial OXPHOS in pancreatic cancer cells [46]. To address the excess of acid products, such as lactate, from aerobic glycolysis, pancreatic cancer cells robustly express monocarboxylate transporter 1 (MCT1), MCT4 and CD147 on the plasma membrane to accelerate lactate flux [47–49]. The nonoxidative pentose phosphate pathway (PPP) originating from glycolysis offers materials for anabolism, including DNA synthesis. In this enhanced metabolic pathway, pancreatic cancer cells display increased ribulose 5-phosphate isomerase (RPIA) and ribulose-5-phosphate-3-epimerase (RPE) expression [50, 51]. The hexosamine biosynthesis pathway (HBP), another branch of glucose metabolism, provides the substrate for protein and lipid glycosylation, which is considered to be critical for tumor progression [52]. In addition, the rate-limiting enzyme of HBP, glutamine:fructose-6-phosphate amidotransferase-1 (GFPT1), is upregulated in pancreatic cancer cells [39]. In addition to those enhanced glycolytic enzymes, pancreatic cancer cells also overexpress more Nampt than adjacent normal tissues. Nampt is an essential enzyme that recycles nicotinamide adenine dinucleotide (NAD), a vital cofactor in many redox reactions, to sustain the high level of glycolytic flux within cancer cells [53]

Such distinct metabolic features are regulated by various factors. KRAS, a kind of small GTPase, is persistently activated upon mutation in pancreatic cancer and continuously stimulates downstream effectors (e.g., PI3K and RAF) [54]. As a result, the expression of *GLUT1* and the key enzymes in glucose metabolism mentioned before are promoted (Fig. 1). Regarding the underlying mechanisms, the KRAS-driven MAPK pathway and transcription factor MYC might be prominent mediators, but the refined regulation of the respective enzymes remains to be further studied [39, 54]. In addition to the regulation of glycolysis, mutant KRAS signaling stimulates mitochondrial translocation of phosphoglycerate kinase 1 (PGK1), leading to phosphorylated PDHK1 and restricted OXPHOS in pancreatic cancer cells [46]. Glucose deprivation also promotes *KRAS* mutations in turn, suggesting the complex interaction between metabolism and the oncogene [55]. Furthermore, some KRAS-driven overexpressed enzymes, such as RPIA, are still preserved in some pancreatic cancer cell lines with KRAS ablation, sustaining nonoxidative PPP and cancer cell survival in a KRAS-independent manner [50]. Therefore, the relationship between KRAS and reprogrammed metabolism needs further study. Additionally, mutant TP53 is a key player in enhancing the Warburg effect and reducing mitochondrial activity [45, 56]. In pancreatic cancer cells, TP53 can increase glucose uptake by increasing paraoxonase 2 expression and impairing the expression of TP53-induced glycolysis and apoptosis regulator (TIGAR), which degrades fructose-2,6-bisphosphate, an effective enhancer of glycolysis, to fructose-6-phosphate [40, 57, 58]. Moreover, mutant TP53 in pancreatic cancer cells also maintains the cytoplasmic stabilization of glyceraldehyde-3-phosphate dehydrogenase, a key enzyme in glycolysis, to support the Warburg effect and confer sensitivity to glycolysis inhibitors (2-deoxyglucose, also known as 2-DG) [59]. However, pancreatic cancer cells with normal TP53 status are resistant to LDHA inhibition due to decreased dependence on glycolysis [57].In addition to KRAS and TP53 signaling in pancreatic cancer cells, LDHA could also be comprehensively regulated by deacetylation modification and other oncogenic transcription factors, such as Forkhead box protein M1 (FOXM1) [60, 61]. Additionally, there are many other mechanisms regulating glucose metabolism in pancreatic cancer cells. For example, hypoxia-inducible factor-1 (HIF-1) mainly induced by hypoxia contributes to strengthened glycolysis and the upregulated expression of HBP-related enzyme (GFPT2, the isoform of GFPT1) [62]. HIF-1 also inhibits PDH, leading to compromised mitochondrial oxidation [63]. Pyruvate kinase muscle isozyme 2 (PKM2) expression in pancreatic cancer cells is largely determined by nutrient conditions, and PKM2 is overexpressed under normal conditions. However, low glucose levels decrease PKM2 expression, which maintains cell survival by promoting autophagy and biomacromolecule accumulation and reducing oxidative stress [64].

#### Amino acids

Amino acid metabolism is also widely rewired in pancreatic cancer cells. Several amino acid transporters are highly expressed in pancreatic cancer cells to satisfy the increased need [65, 66]. Among various amino acids, glutamine (Gln) metabolism is critical for cancer cell survival as the main source of nitrogen and carbon, contributing to macromolecular synthesis and redox balance [67, 68]. Initially, Gln entering mitochondria is deaminated to glutamate (Glu) by glutaminase 1 (GLS1). In many cancer cell lines, aminotransferase or glutamate dehydrogenase (GDH) usually catalyzes the conversion from Gln-derived Glu to α-ketoglutarate (α-KG), which depends on different situations [67]. However, in pancreatic cancer cells, GDH is repressed, but the expression of cytoplasmic aspartate transaminase (GOT1) is promoted [69]. In this process, mitochondrial aspartate transaminase (GOT2) converts Gln-derived Glu and oxaloacetate (OAA) to aspartate (Asp) and α-KG in mitochondria. After that, Asp enters the cytoplasm and is turned into OAA by upregulated GOT1. Then, cytoplasmic OAA is converted to malate through malate dehydrogenase 1 (MDH1) and subsequently oxidized to pyruvate by malic enzyme. In addition, sufficient reducing power is generated to resist reactive oxygen species (ROS) and achieve redox control in pancreatic cancer cells [69]. Consistently, pancreatic cancer cells upregulate the expression of GOT1 in the acidic microenvironment to deal with increased ROS generation and support cancer cell survival [70].

This pathway in pancreatic cancer cells is primarily dominated by mutant KRAS, which results in GDH repression and GOT1 promotion. Therefore, it is named KRAS-driven noncanonical Gln metabolism [69]. Additionally, the regulation of some other processes is also involved in this process. For example, arginine methylation of MDH1 induced by coactivator-associated arginine methyltransferase 1 (CARM1) suppresses tumor growth, but KRAS activation and oxidative stress relieve such inhibition [71]. Furthermore, a recent report revealed that pancreatic cancer cells have compensatory metabolic networks of Gln, exhibiting recovered Gln-derived metabolic intermediates and tumor growth after long-term GLS1 inhibition [72]. In particular, Gln metabolism is vital for the viability and proliferation of hypoxic pancreatic cancer cells, which is mainly mediated by upregulated GLS2, the isoform of GLS1 [62]. Thus, the shift of Gln metabolism in pancreatic cancer is worth further study.

In addition to Gln, other amino acids are also key players in pancreatic cancer progression. Pancreatic cancer cells show overexpressed proline (Pro) oxidase (PRODH1), which contributes to Pro-derived Glu and promotes the survival and proliferation of pancreatic cancer cells, especially under glucose- or Gln-limited conditions [19]. Additionally, many pancreatic tumors from clinical cases display negative or low asparagine (Asn) synthetase expression, indicating the dependence of exogenous Asn. Therefore, plasma Asn depletion mediated by erythrocyte-entrapped L-asparaginase (ERY-ASP) might be a novel therapeutic strategy [73]. Moreover, increased circulating and intracellular branched-chain amino acids are also related to pancreatic cancer progression, which might be the result of enhanced tissue protein breakdown and decreased tumor use mediated by mutant TP53 [38, 56, 74].

Pancreatic cancer also has an extraordinary amino acid degradation ability, possessing urea cycle pathways comparable to those in the liver, which are critical for cancer as well. Obesity or constitutively active AKT, a kinase known to accelerate cancer growth, can induce high expression of arginase 2 that catabolizes arginine into urea and ornithine within the mitochondria of pancreatic cancer cells [75].

#### Lipids

Lipid metabolism is also essential for cancer progression [76]. It not only provides ample building blocks for rapid membrane formation but also produces signaling molecules and substrates for the posttranslational modification of proteins. Lipids can be acquired via biosynthesis and diet. In contrast to normal cells relying on dietary fat, approximately 93% of triacylglycerol fatty acids in tumor cells are de novo synthesized from mitochondrial citrate [77], which is the intermediate between mitochondria and cytosolic acetyl coenzyme A (CoA) [78]. In pancreatic cancer, many enzymes participating in de novo fatty acids and cholesterol synthesis are obviously upregulated, including citrate synthase (CS), ATP citrate lyase (ACLY), fatty acid synthase (FASN), stearoyl-CoA desaturase (SCD1) and 3-hydroxy-3-methylglutaryl coenzyme A reductase (HMGCR) [78, 79] (Fig. 1). Additionally, hypoxia or oncogenic KRAS could also induce the uptake of monounsaturated fatty acids from extracellular lysophospholipids [80]. In de novo cholesterol synthesis, in addition to the elevated canonical pathway, overexpressed aldo-keto reductase 1B10 (AKR1B10) could metabolize farnesal and geranylgeranyl in pancreatic cancer, providing intermediates for cholesterol synthesis [81]. Additionally, those intermediates are also significant for protein prenylation, which activates KRAS and its downstream carcinogenic signaling pathways [81, 82]. Cholesterol acquisition also highly relies on enhanced extracellular uptake in pancreatic cancer cells. Compared with the modestly increased cholesterol synthesis pathway, overactive low-density lipoprotein receptor (LDLR)-mediated uptake of cholesterol-rich lipoproteins is predominant in murine pancreatic cancer cells [79]. After that, excessive free cholesterol is stored as cholesteryl ester within pancreatic cancer cells after esterification, which is mediated by highly expressed acyl-CoA cholesterol acyl-transferase-1 (ACAT-1) [83].

Among different fatty acids, saturated and monounsaturated fatty acids are considered to promote the growth of pancreatic cancer cells [84]. Polyunsaturated fatty acids, mainly containing the ω3 and ω6 families, dually affect pancreatic cancer. ω3 fatty acids inhibit cancer cell proliferation via reducing AKT phosphorylation, but ω6 fatty acids increase AKT phosphorylation [85]. However, a transcriptomics and metabolomics study revealed that lipase and a panel of fatty acids are significantly decreased in pancreatic tumors, and two saturated fatty acids, palmitate, and stearate, showed an obvious ability to inhibit the proliferation of pancreatic cancer cells [86]. Therefore, the role of fatty acids in pancreatic cancer is complicated and still not very clear. Cholesterol also participates in pancreatic cancer progression. Statins, inhibitors of cholesterol de novo synthesis, contribute to improved survival in pancreatic cancer patients in some clinical studies, but the underlying mechanism is still under debate [87]. However, a finished phase II clinical trial combining simvastatin with gemcitabine in advanced pancreatic cancer treatment failed to show clinical benefit (NCT00944463) [88]. Nevertheless, several clinical trials combining statins with other agents in pancreatic cancer treatments are still ongoing (NCT03889795) (NCT03889795). In contrast to the positive role of cholesterol in pancreatic cancer progression, high-level free cholesterol with ACAT-1 inhibition results in severe endoplasmic reticulum (ER) stress and cancer cell apoptosis [83].

### Improving nutrient acquisition by scavenging and recycling

In addition to reprogramming the metabolism of glucose, amino acids and lipids within cells, pancreatic cancer cells have multiple other mechanisms by which they acquire enough fuels for survival and growth.

Macropinocytosis is a process located in the cell membrane that represents bulk extracellular fluid uptake through large endocytic vacuoles, which is crucial for maintaining the amino acid supply of pancreatic cancer cells after subsequent intracellular digestion and degeneration [89, 90]. Oncogenic KRAS plays a key role in promoting macropinocytosis with the help of αvβ3 and galectin-3 on the surface of tumor cells. Moreover, the mutant KRAS/galectin-3/αvβ3 complex also maintains the redox balance of pancreatic cancer cells [91, 92]. Autophagy is another critical cellular process that degrades cellular macromolecules and organelles, affording the recycling of intracellular bioenergetic components. Therefore, it plays a key role in maintaining energy homeostasis and metabolic fuel sources in the tumor [93]. Furthermore, autophagy also enables pancreatic cancer progression via controlling ROS production and sustaining OXPHOS [94]. In turn, elevated ROS promotes autophagy in pancreatic cancer cells [94]. This process is also under the regulation of KRAS. Surprisingly, recent significant studies suggested that the inhibition of KRAS and/or downstream RAF-MEK-ERK signaling pathway could obviously upregulate autophagic flux, which might be the metabolic adaption of compromised glycolysis and mitochondrial activity. Consistent with these findings, combining MEK and autophagy inhibition showed exciting results in both preclinical and clinical studies [95, 96]. TP53 also participates in the regulation of autophagy in pancreatic cancer, but the mechanism is unclear and merits further study [97, 98].

Both macropinocytosis and autophagy undergo degradation in lysosomes to regenerate nutrients. In this process, SLC38A9, an arginine-regulated transporter, facilitates the release of amino acids from lysosomes and activates mechanistic target of rapamycin complex 1 (mTORC1), both of which support pancreatic tumor growth [99]. mTORC1 is a homodimer composed of four mTOR units and a regulatory-associated protein. It is mainly activated by high levels of intracellular amino acids and growth factor signaling and phosphorylates multiple downstream targets and regulates metabolism and tumor progression [100]. Under nutrient-sufficient conditions, activated mTORC1 suppresses autophagy and inhibits the utilization of extracellular proteins through macropinocytosis [101–103]. However, under nutrient-poor circumstances, inactive mTORC1 contributes to the increase in autophagy and macropinocytosis [102–104] to sustain pancreatic tumor growth.

There are several other regulatory relationships involved. For example, MiT/TFE proteins promote the expression of autophagy-lysosome genes with the help of importin 8, regardless of the inhibition from active mTORC1 [105]. Recently, a study suggested that deprivation of amino acids could also induce protein scavenging independently of mTORC1 and that mTOR inhibition could restrict protein synthesis and preserve the intracellular amino acid pool, sustaining the growth of murine pancreatic tumor cells under amino acid deprivation [106].

### Metabolic crosstalk within the microenvironment

**Fig. 2:**
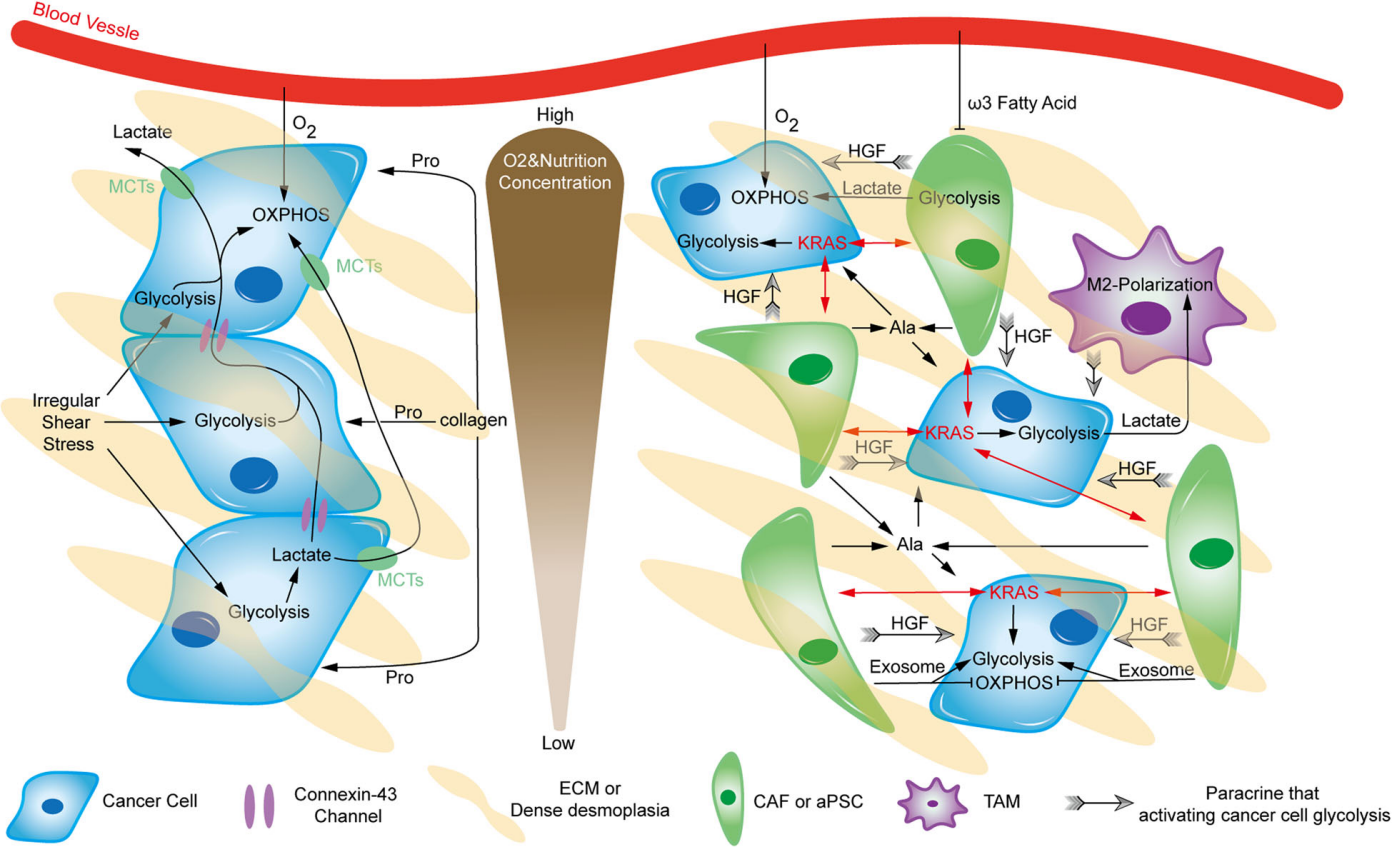
Metabolic crosstalk within the microenvironment. There is extensive and heterogeneous metabolic crosstalk within pancreatic tumors. Pancreatic cancer cells can adopt relatively distinct metabolic patterns under different oxygen and nutrition conditions. Black arrows imply shift, positive regulation or fueling, whereas blunt ends indicate inhibition. Ala, alanine; HGF, hepatocyte growth factor

The pancreatic cancer microenvironment is highly heterogeneous. In addition to cancer cells, ECM and stromal cells are also present. The interest in their interactions in metabolism has been increasing recently [107, 108] (Fig. 2).

Pancreatic cancer cells are surrounded by tight desmoplasia composed of a collagen meshwork, resulting in hypoxic and nutrient-poor conditions, especially in the tumor core. This collagen provides Pro to fuel cancer cells [19]. Additionally, irregular shear stress caused by dense desmoplasia leads to PI3K/AKT signaling upregulation and ROS production, both of which can enhance glycolysis in cancer cells [109]. Furthermore, there are massive amounts of lactate in the tumor, which is distinctly treated by different pancreatic cancer cells under normoxia and hypoxia. Connexin-43 channels, a kind of gap junction, are important for transporting excess lactate from glycolytic pancreatic cancer cells in the tumor core to the periphery, supplying substrates for OXPHOS in better-perfused normoxic cancer cells and producing a suitable chemical milieu for pancreatic tumor growth [110]. Additionally, lactate within the microenvironment can also be sensed by GPR81, a Gi-coupled receptor on the pancreatic cancer membrane, promoting the expression of *MCTs* and *CD147*. In addition, activated GPR81 upregulates peroxisome proliferator-activated receptor gamma coactivator-1α (PGC-1α) and increases mitochondrial biogenesis and respiration. Therefore, both lactate absorption and use are improved, which is especially significant for the growth of pancreatic cancer cells in low glucose conditions [111]. A recent study also revealed that circulating lactate contributes to the TCA cycle of pancreatic tumors as a primary substrate during the fasted state [112]. Additionally, lactate produced by glycolytic cancer cells has some nonmetabolic roles in tumors, such as improving invasiveness, decreasing antitumor immunity and facilitating angiogenesis [37, 113].

Cancer-associated fibroblasts (CAFs), the major type of stromal cells, can be stimulated by neighboring cancer cells, to exhibit aerobic glycolysis and secrete high-energy metabolites, such as pyruvate and lactate. Adjacent cancer cells, particularly in normoxic regions, uptake and use the metabolites in OXPHOS. Such a pattern between CAFs and cancer cells is named the reverse Warburg effect or two-compartment metabolic coupling model, which has attracted considerable attention [44, 114, 115]. Therefore, more recent studies have refocused the effects of OXPHOS in pancreatic cancer cells [116, 117]. CAFs also enrich the microenvironment by releasing exosomes, which contain TCA cycle intermediates, amino acids, and lipids. They are key to pancreatic tumor growth, especially under nutrient-deprived conditions. However, pancreatic cancer cells display suppressed mitochondrial OXPHOS and increased glycolysis upon absorbing these exosomes [118]. The majority of CAFs are derived from pancreatic stellate cells (PSCs), which are activated during carcinogenesis. They can secrete autophagy-derived alanine to support pancreatic cancer cell metabolism, especially after being stimulated by cancer cells [119]. In addition to direct metabolite supply, activated PSCs can be particularly stimulated by oncogenic KRAS in adjacent pancreatic cancer cells, reciprocally enhancing downstream pathways of oncogenic KRAS signaling in cancer cells, including metabolic regulation [120]. Furthermore, enhanced glycolytic metabolism in pancreatic cancer cells can also be induced by paracrine hepatocyte growth factor (HGF) from PSCs [121]. Another recent study indicated that PSCs promote pancreatic cancer progression in a particular manner depending on PKM2 in either cancer cells or PSCs, but the mechanism is unclear and remains to be further studied [122]. In addition, saturated and monounsaturated fatty acids seem to be opposite players in fibrosis and activation of PSCs [123], but their particular roles in activated PSCs of pancreatic cancer are still unknown. Nevertheless, Lipidem^TM^, an ω3 fatty acid-rich emulsion, shows the ability to decrease PSC proliferation and inhibit the invasive capacity of pancreatic cancer cells, especially in combination with gemcitabine [124].

Tumor-associated macrophages (TAMs), another essential type of stromal cell with immune functions, also participate in metabolic crosstalk within the microenvironment. Compared with steady-state macrophages in the normal pancreas, TAMs exhibit an elevated glycolytic signature, promoting pancreatic cancer vascularization and metastasis [125]. Another study showed that TAMs promote aerobic glycolysis in neighboring pancreatic cancer cells via paracrine signaling. Subsequently, lactate in the microenvironment promotes the procancer M2-like polarization of TAMs, which leads to low immunity [126]. Such a relationship was also revealed in preclinical experiments on novel molecules, providing hits for further research. Metavert, an inhibitor of glycogen synthase kinase 3β and histone deacetylases, could normalize the glucose metabolism of pancreatic cancer cells and transform M2-like TAMs to the anticancer M1 phenotype in mouse models [127].

Adipocytes also have extensive metabolic interactions with pancreatic cancer cells. After coculture with pancreatic cancer cells, adipocytes exhibited smaller size, mesenchymal phenotypes, decreased lipid content and multiple altered metabolic pathways. Such tumor-associated adipocytes could also promote the aggressiveness of pancreatic cancer cells [128]. In addition, a brief study in murine cell lines suggested that pancreatic cancer cells could inhibit Gln degeneration in cocultured adipocytes and then predispose them to Gln secretion. In turn, Gln derived from adipocytes facilitates cancer cell proliferation [129]. In addition to the direct interaction between pancreatic cancer cells and adipocytes, adipocyte accumulation within the microenvironment could interact with PSCs and tumor-associated neutrophils as well, enhancing tumor progression, particularly in obese patients [130]. There are many other stromal components besides the abovementioned components, and the metabolic crosstalk within the microenvironment of pancreatic cancer remains largely unclear.

## Chemoresistance and metabolism

**Fig. 3:**
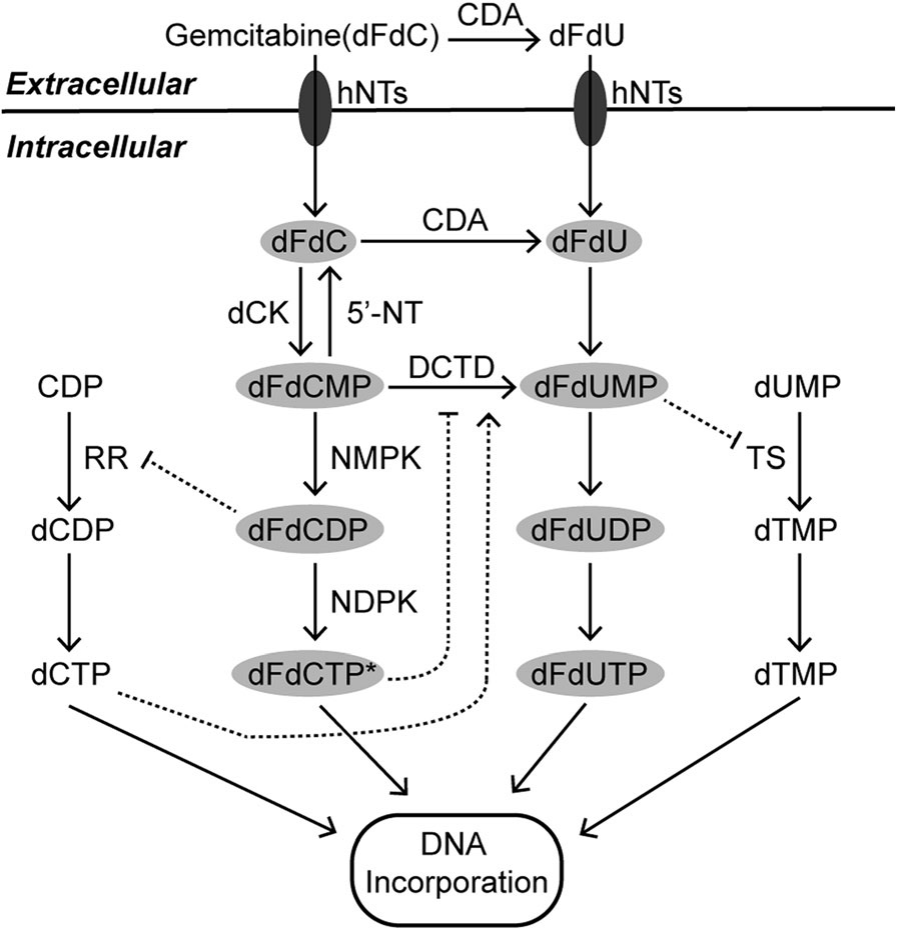
The metabolism and mechanisms of gemcitabine action. Gemcitabine plays an anticancer role after a series of phosphorylations in pancreatic cancer cells. Asterisks indicate that dFdCTP is the most active metabolite of gemcitabine that produces anticancer effects. Intermediates in gray ovals have anticancer functions. Long solid arrows represent shifts or bioconversions. Dotted arrows mean positive regulations, while dotted blunt ends mean negative regulations. dFdC, 2’,2’-difluorodeoxycytidine (gemcitabine); dFdCMP, 2’,2’-difluorodeoxycytidine 5’-monophosphate; dFdCDP, 2’,2’-difluorodeoxycytidine 5’-diphosphate; dFdCTP, 2’,2’-difluorodeoxycytidine 5’-triphosphate; dFdU, 2’,2’-difluorodeoxyuridine; dFdUMP, 2’,2’-difluorodeoxyuridine 5’-monophosphate; dFdUDP, 2’,2’-difluorodeoxyuridine 5’-diphosphate; dFdUTP, 2’,2’-difluorodeoxyuridine 5’-triphosphate; dUMP, deoxyuridine monophosphate; dTMP, deoxythymidine monophosphate; dTTP, deoxythymidine triphosphate; CDP, cytidine diphosphate; dCDP, deoxycytidine diphosphate; dCTP, deoxycytidine triphosphate; CDA, cytidine deaminase; dCK, deoxycytidine kinase; DCTD, deoxycytidylate deaminase; hNTs, human nucleosides transporters; NDPK, nucleoside diphosphate kinase; NMPK, nucleoside monophosphate kinase; RR, ribonucleotide reductase; TS, thymidylate synthase; 5’-NT, 5’-nucleotidase

**Fig. 4:**
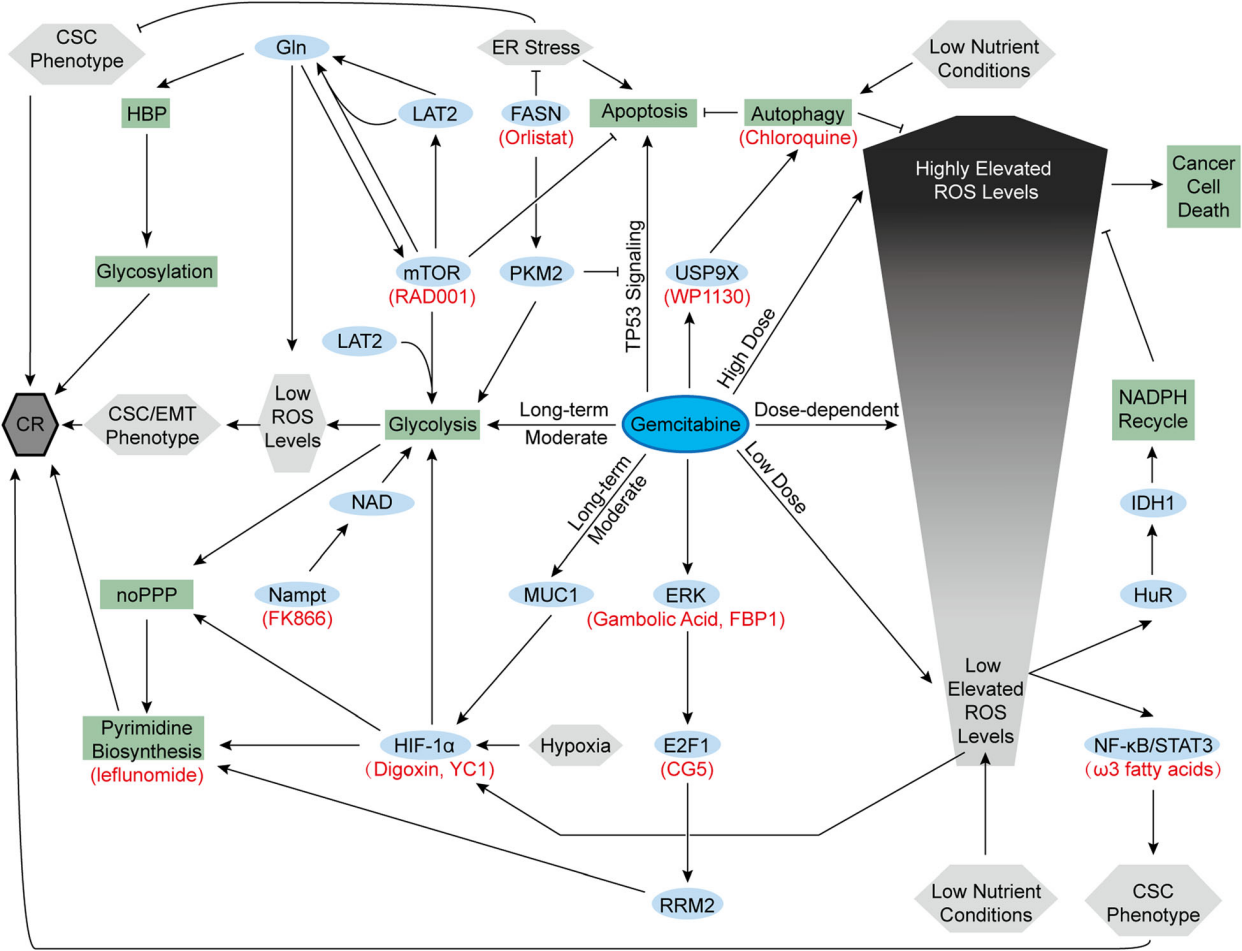
Gemcitabine resistance and metabolism. Gemcitabine and some intrinsic characteristics of pancreatic cancer cells produce chemoresistance. Ovals are biological substances, rectangles are processes, and hexagons are statuses. Red names indicate corresponding inhibitors. Arrows represent positive regulations, and lines with blunt ends represent negative regulations. CR, chemoresistance; EMT, epithelial-mesenchymal transition; FBP1, fructose-1,6-bisphosphatase 1; IDH1, isocitrate dehydrogenase 1; LAT2, L-type amino acid transporter 2; noPPP, nonoxidative pentose phosphate pathway; RRM2, ribonucleotide reductase subunit-M2

Chemotherapy is still the most fundamental systemic treatment against the majority of cancers. According to the NCCN guidelines, there are several distinct chemotherapy regimens against pancreatic cancer with different statuses. Among them, gemcitabine (also known as 2,2-difluorodeoxycytidin, dFdC), the nucleoside analog of deoxycytidine, currently remains the cornerstone of chemotherapy in pancreatic cancer treatments [131]. As a type of prodrug, gemcitabine enters pancreatic cancer cells and undergoes a series of phosphorylation events with precise regulation. After that, its derivatives can interfere with DNA synthesis and block cancer cell cycle progression [132, 133] (Fig. 3). Nano albumin-bound (nab) paclitaxel delivers a high concentration of paclitaxel within pancreatic tumors, resulting in the inhibition of microtubule depolymerization and cancer cell division [134, 135]. Benefiting from the synergistic effects [136, 137], the clinical application of nab paclitaxel often occurs in combination with gemcitabine. In addition to gemcitabine, 5-fluorouracil (5-FU), an analogue of uracil, also exerts anticancer effects by damaging DNA and RNA and inhibiting thymidylate synthase (TS) [138]. Although the clinical benefits produced by 5-FU are lower than those of gemcitabine, 5-FU is still widely applied in treating pancreatic cancer partly due to its lower toxicity [139]. In recent years, increasing evidence has shown that the FOLFIRINOX regimen (5-FU, leucovorin, irinotecan and oxaliplatin) can achieve longer overall survival than gemcitabine-based therapy, especially in patients with good status [140–144]. Compared with other chemotherapy regimens, the underlying mechanism of gemcitabine resistance is relatively well documented [131]. At present, there is increasing evidence that gemcitabine resistance is related to the metabolism of glucose, amino acids, and lipids (Fig. 4). Moreover, metabolic profiling revealed that there is an obvious difference in the metabolome between gemcitabine-sensitive and gemcitabine-resistant pancreatic cancer cell lines [145].

Chemoresistant pancreatic cancer cell lines induced by long-term moderate gemcitabine treatment exhibit increased aerobic glycolysis and lower ROS levels than their parental cells. The increased glycolysis maintains low ROS levels that induce cancer stem cell (CSC) and epithelial-mesenchymal transition (EMT) phenotypes, contributing to chemoresistance [146]. Such enhanced glycolysis is partly mediated by increased HIF-1α. In addition to hypoxia, increased expression of MUC1, a transmembrane protein, also activates and stabilizes HIF-1α, enhancing glycolysis, nonoxidative PPP and pyrimidine biosynthesis [147–149]. All of these factors lead to gemcitabine resistance in pancreatic cancer cells. Owing to this mechanism, HIF-1α inhibitors (digoxin or YC1) and pyrimidine biosynthesis inhibitors (leflunomide) showed the ability to improve gemcitabine efficacy in animal studies [149]. Furthermore, MUC1 inhibition also sensitizes pancreatic cancer cell lines to 5-FU [150]. In addition, F-box and WD repeat domain-containing 7 (FBW7), a pancreatic tumor suppressor inhibited by oncogenic *KRAS* mutation, inhibited glycolysis in pancreatic cancer cells and enhanced the efficacy of gemcitabine in xenograft models as well [151]. In our previous research, we found that L-type amino acid transporter 2 (LAT2), an oncogenic protein in pancreatic cancer cells, could Gln-dependently activate mTOR to inhibit apoptosis and promote glycolysis. Both of them give rise to the gemcitabine-resistant phenotype, whereas mTOR inhibitor (RAD001) could solve such gemcitabine resistance [152]. Overexpressed Nampt provides massive NAD, sustaining enhanced glycolytic activity and contributing to gemcitabine resistance as well. Nampt inhibitor (FK866) reversed this resistance to sensitivity [53]. Additionally, gemcitabine also promotes the expression of the ribonucleotide reductase M2 (RRM2) subunit in pancreatic cancer cells through the ERK/E2F1 pathway, promoting deoxyribonucleotide biosynthesis and inhibiting gemcitabine-induced DNA damage [153, 154]. CG-5, a glucose transporter inhibitor, inhibits E2F1 expression and enhances gemcitabine efficacy in pancreatic cancer cells [153]. Gambogic acid and fructose-1,6-bisphosphatase 1 inhibits the ERK signaling pathway and bypasses gemcitabine resistance in mouse models with xenograft tumors [154, 155]. Increased thymidylate synthase expression in gemcitabine-treated pancreatic cancer cells might also adopt the same E2F1-dependent pathway, but this effect is not very clear yet [153]. In addition to pyrimidine biosynthesis, enhanced HBP in pancreatic cancer cells also gives rise to gemcitabine resistance by increasing the glycosylation of many proteins in several chemoresistant signaling pathways [156].

Compared with the relatively well-documented role of glucose metabolism, the role of amino acid metabolism in chemoresistance remains unclear. Enhanced Gln metabolism fuels elevated HBP and glycosylation. Moreover, Gln addiction is also significant for controlling ROS generation and activating mTOR, both of which contribute to chemoresistance [152, 156]. ROS production in pancreatic cancer cells triggered by gemcitabine treatment is presumed to be related to the dose. Low to moderately elevated ROS levels are considered to activate the nuclear factor-kappa B (NF-κB)/signal transducer and activator of transcription 3 (STAT3) signaling cascade, maintaining the CSC phenotype and inducing chemoresistance [157]. In addition to gemcitabine, low nutrient conditions also contribute to moderate ROS generation that activates HuR, an RNA binding protein. Activated HuR rapidly upregulates isocitrate dehydrogenase 1 (IDH1) to enhance NADPH recycling, which maintains redox balance and results in chemoresistance [158]. Therefore, compared with pancreatic cancer patients with high serum glucose, patients with low or normal serum glucose exhibit more severe initial gemcitabine resistance [158].

In lipid metabolism, FASN expression is upregulated, which is also crucial for gemcitabine resistance. Overexpressed FASN in pancreatic cancer cells upregulates PKM2 expression, promoting glycolysis and gemcitabine resistance [159]. PKM2 also plays a nonmetabolic role in chemoresistance by inhibiting gemcitabine-induced TP53 signaling and subsequent apoptosis [160]. In addition to PKM2, high FASN levels relieve ER stress, maintain the CSC phenotype and inhibit gemcitabine-induced apoptosis. Orlistat, a FASN inhibitor, induces ER stress and increases gemcitabine sensitivity in mouse models with orthotopic pancreatic cancer implantation [161]. ω3 fatty acids repress NF-kβ and STAT3 activation and improve the anticancer role of gemcitabine as well [162]. Furthermore, in a completed phase II clinical trial, after treatment with gemcitabine and intravenous ω3 fatty acid-rich emulsion, patients with metastatic or locally advanced pancreatic cancer exhibited reduced proinflammatory circulating growth factors and cytokines, which might contribute to the improved outcome (NCT01019382) [163]. In addition to fatty acids, cholesterol uptake disruption mediated by LDLR silencing also enhances gemcitabine-induced regression of murine pancreatic cancer cells [79]. Cholesterol also supports the function of caveolin-1 (cav-1) [164], which is the primary structural protein of caveolae, contributing to nab-paclitaxel uptake and chemosensitivity [165]. However, another study suggested that both cholesterol and cav-1 could maintain ABC transporters in caveolae, leading to drug efflux and chemoresistance to nab-paclitaxel in pancreatic tumor initiating cells with high CD133 expression [166]. In general, chemoresistance has intricate relations with the metabolism of pancreatic cancer cells, and further research is needed to achieve better chemotherapy responses.

In addition to the metabolism of glucose, amino acids, and lipids, autophagy in pancreatic cancer cells also plays a role in chemoresistance. Both nutrient limitation and gemcitabine induce autophagy in pancreatic cancer cells, which inhibits apoptosis and contributes to chemoresistance [167, 168]. Gemcitabine-induced autophagy might be mediated by a deubiquitinating protease, ubiquitin-specific peptidase 9X (USP9X). However, WP1130, a deubiquitinating enzyme inhibitor, inhibits USP9X and attenuates chemoresistance in mouse models bearing tumor xenografts [168]. Additionally, chloroquine also increases gemcitabine and 5-FU sensitivity as an autophagy inhibitor [168, 169]. In a completed phase I study, chloroquine showed promising effects in patients with unresectable or metastatic pancreatic cancer when combined with gemcitabine (NCT01777477) [170].

Although there is no evidence showing the direct relationship between metabolic crosstalk within the microenvironment and chemoresistance, the role of the microenvironment in chemoresistance has become more significant. For example, a recent study showed that CAFs in pancreatic cancer could scavenge gemcitabine and contribute to chemoresistance in murine pancreatic cancer [171]. Another study suggested that vitamin D receptors highly expressed on PSCs restrict the tumor-supportive role of PSCs and improve the delivery and efficacy of gemcitabine upon binding ligands [172]. Moreover, an OXPHOS inhibitor (metformin) could overcome CAF-induced chemotherapy resistance and enhance the efficacy of oxaliplatin in pancreatic cancer organoids [173]. In addition to CAFs, nab-paclitaxel internalization of TAMs via macropinocytosis could drive macrophage M1 polarization, restoring immune recognition in pancreatic cancer [174]. Given the extensive and vital metabolic crosstalk within the microenvironment, there might be many potential opportunities to promote the anticancer effects of current chemotherapy.

## Radioresistance and metabolism

In contrast to chemotherapy, there are several controversies regarding the survival benefits of radiotherapy in pancreatic cancer [175–177]. However, it is still an efficient player in controlling the local progression of pancreatic cancer and other solid tumors [178]. According to the National Comprehensive Cancer Network guidelines on pancreatic cancer, radiotherapy is recommended as a neoadjuvant therapy for resectable or borderline disease, an adjuvant therapy for resected disease, a definitive treatment for locally advanced disease, and a palliative care strategy for terminal disease (relieving pain, bleeding and local obstructive symptoms). Additionally, radiotherapy is also recommended for local recurrent pancreatic cancer, but there are limited supporting data. Among many molecular and cellular pathways related to radiotherapy, some clues suggest that metabolic changes in pancreatic cancer are important factors that give rise to radioresistance [179].

Clinical investigations have shown that patients with high baseline metabolism in pancreatic cancer have a poor therapeutic response after receiving chemoradiotherapy [180, 181]. Increased glycolysis-nucleotide metabolism mediated by overexpressed MUC1 in pancreatic cancer also plays a key role in facilitating radioresistance [24]. 2-DG can increase metabolic oxidative stress and cause the radiosensitization of pancreatic cancer by inhibiting glucose metabolism [182]. Ketogenic diets represented by high fat and low carbohydrate intake increased radiotherapy sensitivity in mouse models with xenograft pancreatic cancer. However, a relevant phase I clinical trial in pancreatic cancer patients was not successful, mainly due to poor compliance (NCT01419483) [183]. In addition to glucose metabolism, upregulated FASN likely leads to radioresistance [184, 185]. Several genes involved in the cholesterol synthesis pathway are also associated with radioresistance in pancreatic cancer. Among them, overexpressed farnesyl diphosphate synthase can be inhibited by zoledronic acid (ZOL), which partly attenuated the radioresistance of pancreatic cancer cells [185]. A phase II clinical study combining ZOL with chemoradiotherapy followed by surgery in pancreatic cancer patients is ongoing (NCT03073785). The role of metabolism in pancreatic cancer radioresistance remains to be further researched.

## Immunity and metabolism

Pancreatic cancer is a kind of low immunogenicity tumor that has a highly immunosuppressive microenvironment dominated by three main leukocyte subtypes: TAMs (mainly predominated by M2-type macrophages), regulatory T cells (Tregs) and myeloid-derived suppressor cells (MDSCs) [186, 187]. Emerging evidence suggests that there are close relationships between metabolism and pancreatic cancer immunity, including immunosuppression and immunotherapy resistance.

### Immunosuppression and metabolism

Glucose-dependent metabolism, especially aerobic glycolysis, is critical to fulfilling the immune functions of CD8+ effector T cells and IFN-γ production of CD4+ T cells [188, 189]. In contrast to CD4+ effector T cells (Th1, Th2, and Th17) and M1-type macrophages, Tregs and M2-type macrophages are mainly fueled by lipid oxidation and rely less on glycolysis [190, 191]. Tumor-associated MDSCs also undergo metabolic reprogramming, resulting in both enhanced fatty acid oxidation and increased glycolysis, which sustain their survival and contribute to their immunosuppressive functions [192, 193]. A study in sarcoma revealed that enhancing the glycolysis of tumor cells restricts glucose supply to nearby T cells, thereby leading to dysfunctional T cells and an immunosuppressive tumor microenvironment [25]. In addition to direct nutrition competition, tumor-derived lactate is key to remodeling immunity within the microenvironment, inducing the M2-like phenotype of TAMs and reducing CD8+ cytotoxic T cell functions [194, 195]. More significantly, lactate also upregulates MDSCs and inhibits natural killer cell activity in pancreatic cancer, resulting in an immunosuppressive microenvironment [196]. Additionally, tumor-induced interleukin 6 compromises host metabolism during caloric deficiency, giving rise to suppressed antitumor immunity [20]. In conclusion, metabolism provides novel directions for addressing immunosuppression, which limits the effects of many immunotherapies.

### Immunotherapies and metabolism

Because of the low proportion of resectable cases and obvious resistance to chemotherapy and radiotherapy, immunotherapy has been rising as a novel strategy to treat pancreatic cancer. Many kinds of immunotherapies for pancreatic cancer have entered clinical trial stages, including immune checkpoint inhibitors [11], therapeutic vaccines and adoptive T cell transfers [197, 198]. However, most results are disappointing. Recently, accumulated studies have suggested that T cell-mediated immunotherapy could be optimized by modulating cell metabolism [199]. Moreover, immune checkpoint inhibitors also show the ability to support the metabolism of lymphocytes in the tumor and improve their antitumor effects [25, 200]. Given the relationship between metabolism and immunotherapy, it is appropriate to improve conventional immunotherapies through metabolic regulation.

In addition to immunotherapies focusing on improving the anticancer abilities of lymphocytes, the immunosuppressive microenvironment is emerging as a novel therapeutic target [201]. In addition to leukocyte subtypes, some cytokines also participate in immunosuppression of pancreatic cancer. For example, indoleamine 2,3-dioxygenase (IDO), a metabolic enzyme expressed in many carcinomas, including pancreatic cancer cells, degrades tryptophan within the tumor microenvironment and inhibits immune cell responses [202, 203]. Furthermore, combining IDO depletion and tumor desmoplasia inhibition showed successful antitumor effects in mouse models with pancreatic cancer [204]. Recently, a phase I/II trial combining IDO inhibitor (indoximod) and chemotherapy in patients with metastatic pancreatic cancer was completed (NCT02077881), while another phase II clinical study employing another IDO inhibitor (epacadostat) and immunotherapy or cyclophosphamide in pancreatic cancer patients is recruiting (NCT03006302). In general, given the significant role of metabolism in immunity, metabolic regulation has the potential to improve the clinical results of immunotherapies.

## Clinical perspectives and conclusion

Metabolism targeted therapy is not yet recommended as regular treatment in most guidelines for treating variety of cancers. In addition to the wide use of aromatase inhibitors in treating breast cancer [205], most metabolism-targeted therapies against a variety of cancers remain in experimental and clinical trial phases. However, some of them have shown promising results. For instance, ivosidenib, an inhibitor of IDH1, improves the complete remission rate of IDH1-mutated acute myeloid leukemia with a low frequency of treatment-related adverse events in a phase I clinical trial that enrolled 258 patients [206]. In a phase IV clinical trial that enrolled 28 patients, the mechanism of diclofenac in effectively relieving actinic keratosis (a premalignant skin lesion) was well demonstrated, and the effect was largely dependent on modulating the metabolism of local lesions [207]. However, given the heterogeneity of different cancers, successful clinical applications in other cancers cannot be directly and simply applied to treating pancreatic tumors. In pancreatic cancer, many metabolic regulators have been employed in preclinical studies [208] and even in clinical trials [28, 34]. Compared with some old drugs, such as metformin [209], aspirin [210, 211], and statins, which play complex roles in metabolic regulation and have been debated over a dozen years, there are higher expectations for some new properties of other drugs, novel drug combinations and new metabolic regulators. In addition to the clinical trials mentioned above, many other clinical studies have also focused on metabolism in pancreatic cancer treatments (Table 1).

**Table 1:** Representative clinical trials concerning metabolic regulation in pancreatic cancer

NCT Number	Status	Phase	Tumor Types	Interventions	Monotherapy/Combination
Targets: Glycolysis and Mitochondrial Metabolism					
NCT00096707	Completed	I	Lung Cancer Breast Cancer Pancreatic Cancer Head and Neck Cancer Gastric Cancer	2-DG ± Docetaxel	Monotherapy/Combination
NCT01835041	Active, Not Recruiting	I	Acinar Cell Adenocarcinoma of the Pancreas, Duct Cell Adenocarcinoma of the Pancreas, Recurrent Pancreatic Cancer, Stage IV Pancreatic Cancer	CPI-613 + mFOLFIRINOX	Combination
NCT01419483	Terminated	N/A	Pancreatic Neoplasms	Ketogenic Diet	N/A
Targets: Amino Acids Metabolism and Redox Balance					
NCT02514031	Recruiting	I	Pancreatic Cancer	ARQ-761 + Gemcitabine + Nab-paclitaxel	Combination
NCT01523808	Completed	I	Pancreatic Cancer	GRASPA	Monotherapy
NCT02195180	Completed	II	Metastatic Pancreatic Adenocarcinoma	ERY001 + Gemcitabine or FOLFOX	Combination
NCT02077881	Completed	I/II	Metastatic Pancreatic Adenocarcinoma, Metastatic Pancreatic Cancer	Indoximod + Gemcitabine + Nab-paclitaxel	Combination
NCT03006302	Recruiting	II	Metastatic Pancreatic Adenocarcinoma	Epacadostat + Pembrolizumab + CRS-207 ± Cyclophosphamide/GVAX	Combination
NCT01049880	Completed	I	Pancreatic Neoplasms	Gemcitabine + Ascorbic Acid	Combination
Target: Lipids Metabolism					
NCT00944463	Completed	II	Pancreatic Cancer	Gemcitabine + Simvastatin	Combination
NCT01019382	Completed	II	Pancreatic Neoplasms	Gemcitabine + Lipidem Fish Oil Infusion	Combination
NCT03073785	Recruiting	II	Pancreatic Adenocarcinoma Recurrent Pancreatic Carcinoma Stage I Pancreatic Cancer AJCC v6 and v7 Stage IA Pancreatic Cancer AJCC v6 and v7 Stage IB Pancreatic Cancer AJCC v6 and v7 Stage II Pancreatic Cancer AJCC v6 and v7 Stage IIA Pancreatic Cancer AJCC v6 and v7 Stage IIB Pancreatic Cancer AJCC v6 and v7 Stage III Pancreatic Cancer AJCC v6 and v7 Stage IV Pancreatic Cancer AJCC v6 and v7	Zoledronic Acid + Capecitabine + Fluorouracil + Radiation Therapy	Combination
Targets: Autophagy and Macropinocytosis					
NCT01777477	Completed	I	Pancreatic Cancer	Gemcitabine + Chloroquine	Combination
NCT03825289	Recruiting	I	Metastatic Pancreatic Carcinoma Stage II Pancreatic Cancer Stage IIA Pancreatic Cancer Stage IIB Pancreatic Cancer Stage III Pancreatic Cancer Stage IV Pancreatic Cancer Unresectable Pancreatic Carcinoma	Hydroxychloroquine + Trametinib	Combination
Target: mTOR					
NCT00409292	Completed	II	Pancreatic Cancer	RAD001	Monotherapy
NCT00593008	Terminated	I	Pancreatic Adenocarcinoma	Gemcitabine + Temsirolimus	Combination
NCT01079702	Unknown	I/II	Advanced Malignancies (Including Pancreatic Cancer)	Capecitabine + Everolimus	Combination
Comprehensive Metabolic Regulation					
NCT03889795	Recruiting	I	Advanced Pancreatic Cancer Advanced Solid Tumor	Metformin + Simvastatin + Digoxin	Combination
NCT02201381	Recruiting	III	Cancer (Including Pancreatic Cancer)	Metabolic Treatment (Metformin + Atorvastatin + Doxycycline + Mebendazole)	Combination
NCT02048384	Active, Not Recruiting	I/II	Metastatic Pancreatic Adenocarcinoma	Metformin ± Rapamycin	Monotherapy/Combination

Therapies targeting altered glycolysis pathways are gaining momentum. In a phase I clinical trial in patients with solid tumors, including advanced pancreatic cancer, 2-DG produced tolerable adverse effects but tangible clinical benefits in combination with docetaxel (NCT00096707) [212]. Although PDH is inhibited by mutant KRAS [46], cancer cell survival still requires the persistence of PDH activity and mitochondrial metabolism. CPI-613, a lipoate analog, selectively inhibits tumor PDH activity and could disrupt pancreatic cancer growth in xenograft models [213]. An ongoing phase I study combining CPI-613 with modified FOLFIRINOX for treating metastatic pancreatic cancer showed a small increase in side effects and toxicity compared to that with FOLFIRINOX alone but an encouraging response rate (NCT01835041) [214].

For the noncanonical Gln metabolism of pancreatic cancer, GLS1 inhibition decreases antioxidant pools, whereas β-lapachone induces excess ROS generation specifically in pancreatic cancer cells as an NAD(P)H: quinone oxidoreductase 1-bioactivatable drug. Combining β-lapachone (ARQ761) with GLS1 inhibitors selectively leads to pancreatic cancer cell death in preclinical mouse models [215]. At present, a phase I clinical study that combined ARQ761 with gemcitabine/nab-paclitaxel in patients with advanced pancreatic cancer is ongoing (NCT02514031). For asparagine metabolism, ERY-ASP showed good tolerance in patients with metastatic pancreatic cancer in a phase I clinical study (NCT01523808) [216], and a further phase II clinical trial combining ERY-ASP with chemotherapy was completed recently (NCT02195180).

Given the comprehensive role of mTOR in metabolism, some clinical trials have employed mTOR inhibitors to address gemcitabine resistance in pancreatic cancer. However, RAD001 (everolimus) showed minimal clinical activity as a single agent in patients with metastatic and gemcitabine-resistant pancreatic cancer (NCT00409292) [217], whereas another phase II study combining everolimus with capecitabine showed moderately positive results and an acceptable toxicity profile (NCT01079702) [218]. Therefore, further clinical trials are anticipated.

The rewired glucose, amino acid, and lipid metabolism in pancreatic tumors from the cell to the microenvironment or even at the whole-body level deeply affects cancer progression. Furthermore, pancreatic cancer metabolism is also associated with anticancer treatments. Currently, more clinical trials in pancreatic cancer patients are beginning to involve metabolic regulation. Some of the completed trials even showed promising and exciting results. Given the side effects of some metabolic regulators [219], the role of metabolism targeted therapy in nonmalignant tissues should also be emphasized in future research, which largely restricts the transformation from basic study to successful clinical application. Moreover, pancreatic cancer displayed highly plastic metabolism, suggesting that cancer cells can adapt to use other metabolic pathways to bypass a certain metabolic inhibition [220]. In addition, pancreatic cancer cell lines also showed heterogeneous metabolic addictions [43]. Therefore, identification of the real metabolic hub of pancreatic cancer or combining distinct metabolism-targeted therapies in clinical trials is in high demand. In conclusion, a better understanding of pancreatic cancer metabolism and its role in treatments will benefit novel strategies, improving the prognosis of patients with pancreatic cancer.

## Data Availability

Not applicable.

## Abbreviations

2-DG: 2-deoxyglucose
5-FU: 5-fluorouracil
ACAT-1: Acyl-CoA cholesterol acyl-transferase-1
ACLY: ATP citrate lyase
AKR1B10: Aldo-keto reductase 1B10
Asn: Asparagine
Asp: Aspartate
CAFs: Cancer-associated fibroblasts
CARM1: Coactivator-associated arginine methyltransferase 1
cav-1: caveolin-1
CS: citrate synthase
CSC: Cancer stem cell
ECM: Extracellular matrix
EMT: Epithelial-mesenchymal transition
ER: Endoplasmic reticulum
ERY-ASP: Erythrocyte-entrapped L-asparaginase
FASN: Fatty acid synthase
FBW7: F-box and WD repeat domain-containing 7
FOXM1: Forkhead box protein M1
GDH: Glutamate dehydrogenase
GFPT1: Glutamine:fructose-6-phosphate amidotransferase-1
Gln: Glutamine
GLS1: Glutaminase 1
Glu: Glutamate
GLUT1: Glucose transporter 1
GOT1: Cytoplasmic aspartate transaminase
GOT2: Mitochondrial aspartate transaminase
HBP: Hexosamine biosynthesis pathway
HGF: Hepatocyte growth factor
HIF-1: Hypoxia-inducible factor-1
HMGCR: 3-hydroxy-3-methylglutaryl coenzyme A reductase
IDH1: Isocitrate dehydrogenase 1
IDO: Indoleamine 2,3-dioxygenase
LAT2: L-type amino acid transporter 2
LDH: Lactate dehydrogenase
LDLR: Low-density lipoprotein receptor
MCT1: Monocarboxylate transporter 1
MDH1: Malate dehydrogenase 1
MDSCs: Myeloid-derived suppressor cells
mTORC1: mechanistic target of rapamycin complex 1
MUC1: Mucin1
NAD: Nicotinamide adenine dinucleotide
NF-κB: Nuclear factor-kappa B
OAA: Oxaloacetate
OXPHOS: Oxidative phosphorylation
PDH: Pyruvate dehydrogenase
PDHK1: Pyruvate dehydrogenase kinase 1
PGC-1α: Peroxisome proliferator-activated receptor gamma coactivator-1α
PGK1: Phosphoglycerate kinase 1
PKM2: Pyruvate kinase muscle isozyme 2
PPP: Pentose phosphate pathway
Pro: Proline
PRODH1: Proline oxidase
PSCs: Pancreatic stellate cells
ROS: Reactive oxygen species
RPE: Ribulose-5-phosphate-3-epimerase
RPIA: Ribulose 5-phosphate isomerase
RRM2: Ribonucleotide reductase M2
SCD1: Stearoyl-CoA desaturase
STAT3: Signal transducer and activator of transcription 3
TAMs: Tumor-associated macrophages
TIGAR: TP53-induced glycolysis and apoptosis regulator
Tregs: Regulatory T cells
TS: Thymidylate synthase
USP9X: Ubiquitin-specific peptidase 9X
ZOL: Zoledronic acid
α-KG: α-ketoglutarate
